# Identification of immune-related hub genes and miRNA-mRNA pairs involved in immune infiltration in human septic cardiomyopathy by bioinformatics analysis

**DOI:** 10.3389/fcvm.2022.971543

**Published:** 2022-09-20

**Authors:** Jingru Li, Guihu Sun, Haocheng Ma, Xinyu Wu, Chaozhong Li, Peng Ding, Si Lu, Yanyan Li, Ping Yang, Chaguo Li, Jun Yang, Yunzhu Peng, Zhaohui Meng, Luqiao Wang

**Affiliations:** ^1^Department of Cardiology, The First Affiliated Hospital of Kunming Medical University, Kunming, China; ^2^Department of Emergency, The First Affiliated Hospital of Kunming Medical University, Kunming, China

**Keywords:** septic cardiomyopathy, miRNA, immune-related gene, biomarker, miRNA-mRNA regulatory network

## Abstract

**Abstract:**

Septic cardiomyopathy (SCM) is a serious complication caused by sepsis that will further exacerbate the patient's prognosis. However, immune-related genes (IRGs) and their molecular mechanism during septic cardiomyopathy are largely unknown. Therefore, our study aims to explore the immune-related hub genes (IRHGs) and immune-related miRNA-mRNA pairs with potential biological regulation in SCM by means of bioinformatics analysis and experimental validation.

**Method:**

Firstly, screen differentially expressed mRNAs (DE-mRNAs) from the dataset GSE79962, and construct a PPI network of DE-mRNAs. Secondly, the hub genes of SCM were identified from the PPI network and the hub genes were overlapped with immune cell marker genes (ICMGs) to further obtain IRHGs in SCM. In addition, receiver operating characteristic (ROC) curve analysis was also performed in this process to determine the disease diagnostic capability of IRHGs. Finally, the crucial miRNA-IRHG regulatory network of IRHGs was predicted and constructed by bioinformatic methods. Real-time quantitative reverse transcription-PCR (qRT-PCR) and dataset GSE72380 were used to validate the expression of the key miRNA-IRHG axis.

**Result:**

The results of immune infiltration showed that neutrophils, Th17 cells, Tfh cells, and central memory cells in SCM had more infiltration than the control group; A total of 2 IRHGs were obtained by crossing the hub gene with the ICMGs, and the IRHGs were validated by dataset and qRT-PCR. Ultimately, we obtained the IRHG in SCM: THBS1. The ROC curve results of THBS1 showed that the area under the curve (AUC) was 0.909. Finally, the miR-222-3p/THBS1 axis regulatory network was constructed.

**Conclusion:**

In summary, we propose that THBS1 may be a key IRHG, and can serve as a biomarker for the diagnosis of SCM; in addition, the immune-related regulatory network miR-222-3p/THBS1 may be involved in the regulation of the pathogenesis of SCM and may serve as a promising candidate for SCM therapy.

## Introduction

Septic cardiomyopathy (SCM) is septic myocardial damage caused by severe sepsis and is an important cause of high mortality in non-cardiac intensive care units (1). The current general understanding of SCM is acute cardiac dysfunction caused by a dysregulated host response to infection (2). In addition, the presence of SCM in sepsis patients will induce/aggravate circulatory dysfunction and further deteriorate peripheral organ function, resulting in the mortality rate of patients up to 70% (3). Therefore, in-depth research on septic cardiomyopathy is very necessary.

Cardiomyocyte injury and myocardial contractile dysfunction are the main pathophysiological processes in SCM. The current clinical diagnosis of SCM can only rely on myocardial injury factors and echocardiography, which limits early intervention in SCM patients (4–8). Therefore, it is crucial to select appropriate predictive biomarkers for early intervention in SCM.

MicroRNAs (miRNAs) are a class of transcripts without protein-coding functions and play a regulatory role in inflammation (9), growth and development, and biological metabolism in eukaryotes (10). They can regulate gene expression by promoting messenger RNA (mRNA) degradation or inhibiting the mRNA post-transcriptional process (11, 12). With the development of high-throughput gene expression profiling technology, microarray analysis has been widely used to monitor the abnormal expression of genes or miRNAs in diseases (13). More importantly, circulating miRNAs are proposed as biomarkers for disease diagnosis and monitoring in inflammatory heart disease and sepsis-induced cardiac insufficiency (14, 15).

In SCM, abnormal/excessive immune responses are also considered to be one of the important causes of cardiomyocyte dysfunction. Previous studies have shown that interleukin-1β (IL-1β), activated by NLRP3 inflammasome, impairs cardiac function and causes cardiogenic atrophy and diastolic dysfunction in patients with sepsis (16). Activation of the cholinergic anti-inflammatory pathway (CAP) central alpha7 nicotinic acetylcholine receptor (alpha7nAChR) reverses immunosuppression and attenuates multiorgan dysfunction in T lymphocytes from septic rats (17). This suggests that immune response is an important process in myocardial injury in SCM. However, the exact mechanisms of the immune response and IRGs in SCM myocardial injury remain unclear.

**Figure 1:**
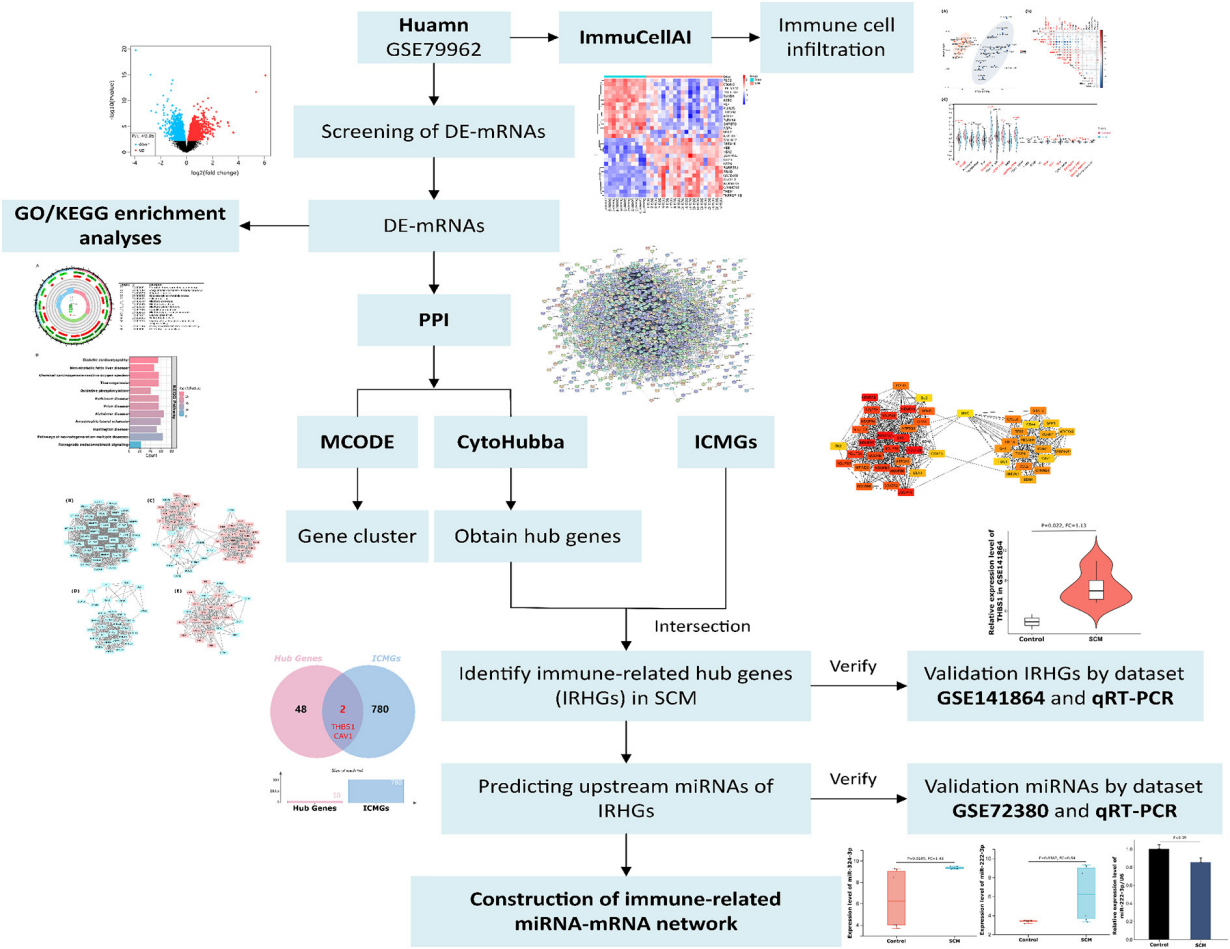
The overall workflow of this study. De-mRNAs: Differentially expressed mRNAs; GO: Gene Ontology; KEGG: Kyoto Encyclopedia of Genes and Genomes; ICMGs: immune cell marker genes.

Therefore, to determine the role of immune responses in SCM, we obtained DE-mRNAs in datasets GSE79962 by bioinformatics analysis. The ImmucellAI tool was used to analyze the differences in immune cell infiltration between the SCM and control samples of the dataset GSE79962. Subsequently, GO/KEGG enrichment analysis was performed on the DE-mRNAs. Based on the STRING online database, we constructed a protein-protein interaction (PPI) network for DE-mRNA and used Cytoscape-MCODE and CytoHubba plugins to identify significant clusters and central genes in the PPI. Then, we overlapped the hub genes with immune cell marker genes (ICMGs) to obtain immune-related hub genes (IRHGs) during SCM. qRT-PCR and GEO datasets were used to validate the expression of IRHGs. Not only that, after screening the target IRHGs, we predicted the upstream miRNAs of IRHGs from the relevant database. After dataset validation, the key immune-related miRNA-IRHG pair was constructed. We summarize the overall workflow of this study and present it in Figure 1.

## Materials and methods

### Expression profile of mRNA and miRNA in microarray data from human and mice with septic cardiomyopathy

Three human and mice septic cardiomyopathy datasets (GSE79962, GSE141864 and GSE72380) were obtained from the Gene Expression Omnibus (GEO) (https://www.ncbi.nlm.nih.gov/geo/) database. Datasets GSE79962 and GSE141864 analyzed mRNA transcript changes in cardiac tissue from patients with septic cardiomyopathy, and dataset GSE72380 collected cardiac tissue from septic mice for miRNA microarray sequencing. The detailed data of these SCM datasets are presented in Table 1. Therefore, we used these 3 datasets to screen and identify hub genes and hub gene-related miRNA-mRNA regulatory networks. The overall flow chart of this study is shown in Figure 1.

**Table 1:** Information on selected microarray datasets.

**GEO accession**	**Experiment type**	**Species**	**Experimental model**	**Source tissue**	**Sample**		**Data**	**Attribute**
					**Control**	**SCM**		
GSE79962	Array	Human	Sepsis	Heart	11	20	mRNA	Test set
GSE141864	Array	Human	Sepsis	Heart	2	8	mRNA	Validation set
GSE72380	Array	Mice	LPS-induced	Heart	6	6	miRNA	Validation set

LPS, lipopolysaccharide.

### Immune cell infiltrate analyses

ssGSEA enrichment analysis is a method for assessing immune cell infiltration in samples in a dataset based on the expression levels of immune cell-specific marker genes. ImmuCellAI (ImmuCellAI, https://bioinfo.life.hust.edu.cn/web/ImmuCellAI/) (18) is an online tool that uses ssGSEA enrichment analysis to investigate immune cell infiltration in expression matrices. Upload the dataset's gene expression matrix to ImmuCellAI and compare it to the defined immune cell-related gene expression matrix, evaluate the quantity of 24 various types of immune cells in the samples, and compare immune cell infiltration in different samples. Then cluster analysis of immune cell infiltration matrix data, and draw a two-dimensional PCA cluster diagram. The correlation heat map shows the correlation of genes in 24 types of immune cell infiltration, and the violin plot shows the difference in immune cell infiltration.

### Differentially expressed transcript analysis

The GEO2R (http://www.ncbi.nlm.nih.gov/geo/geo2r/) tool is an R-based web application for identifying differentially expressed mRNAs (DE-mRNAs) and miRNAs (DE-miRNAs) between SCM and control groups. A *p*-value < 0.05 and an absolute fold-change (|FC|) > 2.5 as the threshold for screening DE-mRNAs and DE-miRNAs. We use a volcano plot to show the overall expression and distribution of genes in the dataset GSE79962, and a heatmap to show the intergroup expression of the top 30 DE-mRNAs.

### Functional enrichment analyses

To understand the characteristic biological properties of DE-mRNAs, the Metascape bioinformatics resource (Metascape, a gene annotation and analysis resource, http://metascape.org, version 3.5) (19) was used for functional enrichment analysis. A minimum overlap value of 3, *p*-value cutoff of 0.05 and minimum enrichment of 1.5 were set as thresholds. The DE-mRNAs screened in dataset GSE79962 were imported into Metascape to evaluate enriched biological process (BP), molecular function (MF) and cellular component (CC), and Kyoto Encyclopedia of Genes and Genomes (KEGG) pathways, and used circle diagram and histograms visualize the enrichment results for GO and KEGG.

### Protein interaction analysis and acquisition of key gene clusters

Protein-protein interaction (PPI) networks have been generated from the STRING database (https://string-db.org/) (20). A rating of 0.4 (medium confidence) used to be set as a threshold. The number of nodes and edges of the PPI network is also obtained from STRING. Next, in order to obtain the gene clusters in the PPI network, the Cytoscape-Minimal Common Oncology Data Elements (MCODE) was used to identify significant gene clusters and obtain cluster scores (filter criteria: degree cut-off = 2; node score cut-off = 0.2; *k*-core = 2; max depth = 100) and visualize it in Cytoscape. The method of using MCODE to identify key genes of the network is recognized, and scholars have used this method to conduct related research (21).

### Acquisition of SCM hub genes and SCM immune-related hub genes, and determination of target genes

CytoHubba is a plugin that can measure the importance of nodes in the network based on network characteristics. The Maximal Clique Centrality (MCC) algorithm was used in CytoHubba to filter the top 50 genes of the PPI network (22). Then, to further elucidate whether these 50 hub genes have immunomodulatory roles, we obtained 782 immune cell marker genes (ICMGs) from published articles (23), and crossed 50 hub genes with ICMGs to obtain hub genes with immunomodulatory effects in SCM, namely immune-related hub genes (IRHGs). IRHGs was further validated using dataset GSE141864 to identify target genes. This analysis of immune-related genes associated with diseases is practical and has been used by scholars for related studies. It is worth emphasizing that this analysis of immune-related genes associated with diseases is practical and has been used by scholars for related studies (24).

### SCM sample collection and real-time quantitative reverse transcription-polymerase chain reaction analysis

Peripheral blood mononuclear cells (PBMCs) samples of patients with septic cardiomyopathy were collected from the Department of Emergency Medicine of the First Affiliated Hospital of Kunming Medical University, Yunnan Province, and medical information was collected by the Declaration of Helsinki after informed consent of the patients. This study was approved by the Ethics Committee of the First Affiliated Hospital of Kunming Medical University, Yunnan Province [(2022), ethical review L, No.23]. Male patients with a definite history of sepsis and a reduced left ventricular ejection fraction (LVEF) <0.5 and a confirmed diagnosis of septic cardiomyopathy were included in the study. Exclusion criteria were patients with: (i) a combination of severe arrhythmias; (ii) a combination of significant organ failure, including liver and kidney; and (iii) the presence of possible myocardial suppression disease. Three healthy males matched to the age of the SCM patients served as controls. Total RNA was isolated using TRIzol reagent (Invitrogen) followed by amplification grade (Invitrogen) DNase 1 for further processing. SuperScript™ III First-Strand Synthesis SuperMix (Cat. No. 11752050; Thermo Fisher Scientific Inc.) for reverse transcription of target genes. Target gene determination was performed using the StepOnePlus™ Real-Time PCR System (Cat. No. 4376600; Thermo Fisher Scientific Inc.) with GAPDH as an internal reference. To quantify miRNA expression, we used an optimized qRT-PCR assay based on SYBR green. Data were analyzed using the 2^−Δ*ΔCt*^ method, with U6 (NR_004394.1) as an internal control for target genes, respectively. The sequences and other characteristics of the primers used in the qRT-PCR analysis are shown in **Table 3**.

### Diagnostic analysis of target genes in SCM

The receiver operating characteristic (ROC) curve was obtained by a “rating” method and a mathematical prediction method based on patient characteristics while meeting statistical calculation criteria. ROC curves were generated by analyzing the expression of markers with 1-specificity as the horizontal coordinate and sensitivity as the vertical coordinate. The area under the curve (AUC) of the ROC curve shows the probability that a randomly selected diseased subject will be (correctly) rated or ranked compared to a randomly selected non-diseased subject. In clinical applications, ROC curves are commonly used to assess the ability of biomarkers to distinguish between patients and non-patients with a given disease. Therefore, to elucidate the diagnostic value of target genes in SCM, ROC curve analysis was used in this study. AUC > 0.5 indicates that the marker has the ability to discriminate between healthy controls and patients with SCM (25, 26).

### Construction of the miRNA-MRNA regulatory network

miRDB (http://mirdb.org) (27), TargetScan (https://www.targetscan.org/vert_80/), StarBase (http://starbase.sysu.edu.cn/) (28) and RNA22 (https://cm.jefferson.edu/rna22/) are four online public databases that collect relevant miRNA-mRNA interaction information. For miRNA target gene prediction, we used miRDB, TargetScan, and StarBase. Then, the miRNA prediction results of the three databases were intersected and further verified in the dataset GSE72380 to obtain the target miRNA. RNA22 was used to reverse predict the target genes of miRNAs for validation of the results. The expression levels of target miRNAs in peripheral blood mononuclear cells of SCM patients were detected by qRT-PCR. Violin plots were used to visualize miRNA expression.

### Statistical analysis of microarray data

The Gene Expression Omnibus (GEO, http://www.ncbi.nlm.nih.gov/geo/) - GEO2R (http://www.ncbi.nlm.nih.gov/geo/geo2r/) was used to identify DEGs in this study. GEO2R is an online application based on the R language. After the user defines the group, GEO2R directly reads the original series matrix file and platform annotation file uploaded by the submitter and draws the boxplot and gene table (including *P*-values, t-statistics and fold changes, and gene annotations including gene symbols, gene names, gene ontology (GO) entries and chromosome locations, etc.) of the expression value distribution of the selected samples through the “boxplot” and “limma” *R* language scripts. *P* < 0.05, FC > 2.5 or FC < 0.4 was set as the threshold for screening differentially expressed genes (29).

## Result

### The landscape of immune cell infiltration in septic cardiomyopathy

**Figure 2:**
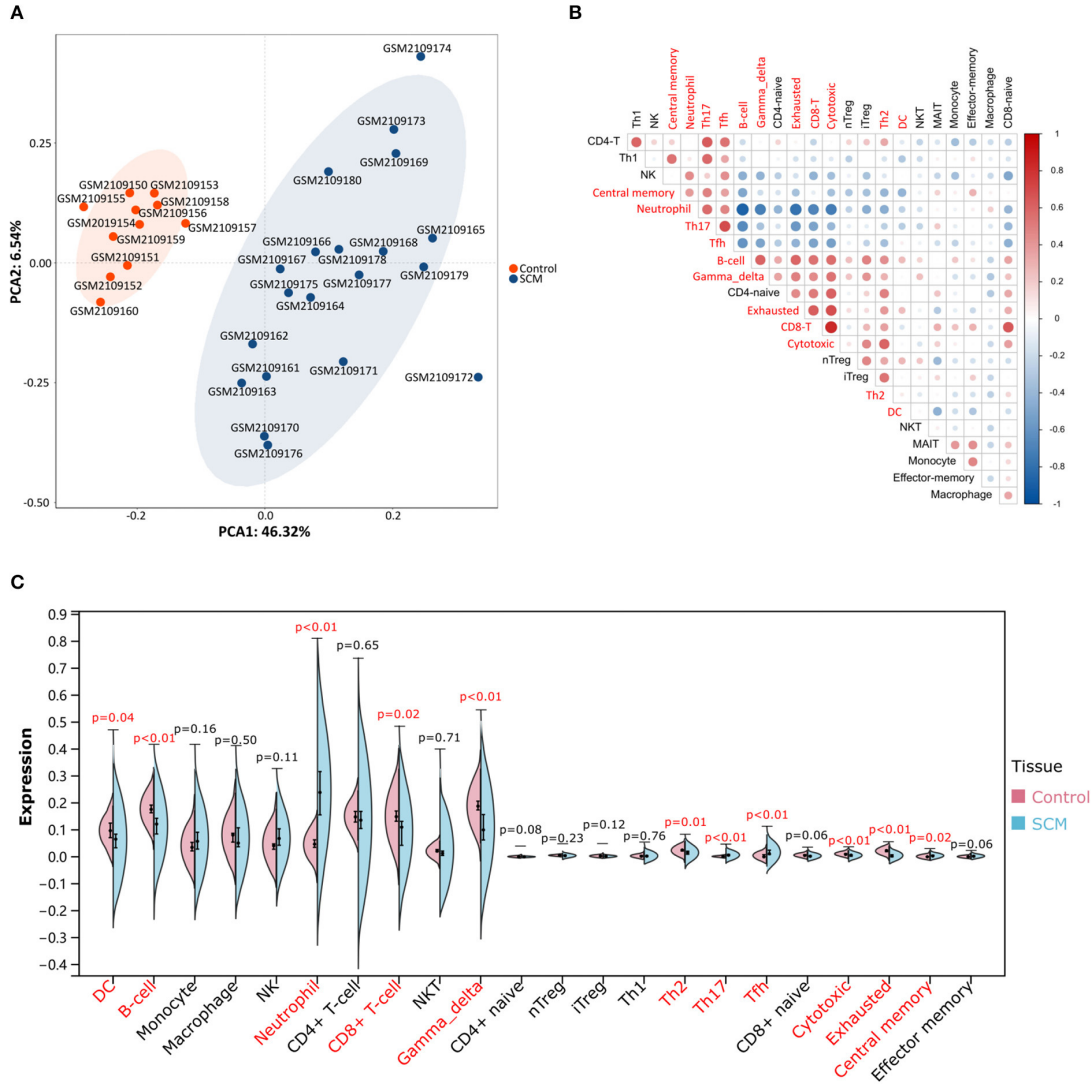
Evaluation and visualization of immune cell infiltration. **(A)** PCA cluster plot of immune cell infiltration between SCM samples and control samples. **(B)** Correlation heatmap of 24 immune cells. The size of the circle represents the strength of the correlation: the darker the color, the stronger the correlation; the color represents the correlation: blue represents a positive correlation, and blue represents a negative correlation. Markers in red font represent differences in the infiltration of this cell type between the two groups. **(C)** Violin plot of the proportion of 24 immune cells. Markers in red font indicate differences in immune infiltration of this cell type between the SCM group and the control group.

PCA cluster analysis is a method for examining the consistency of biological repetition as well as group differences. PCA cluster analysis results of immune cell infiltration showed that gene expression levels in SCM samples and control samples were significantly different (Figure 2A). The correlation heatmap of 24 immune cells showed a significant positive correlation between CD8^+^ T and cytotoxic cells, T helper type 17 (Th17) cells and T follicular helper (Tfh) cells, B cells (B-cells) and gamma delta T cells and exhausted cells, exhausted cells and CD8^+^ T and cytotoxic cells. Significant negative correlations were found between neutrophils and B cells and depleting and cytotoxic cells, Th17 and B-cells, Tfh and B-cells (Figure 2B). The violin plot of the immune cell infiltration difference showed that, compared with the control sample, neutrophil cells, Th17 cells, Tfh cells and central-memory cells infiltrated more and exhausted cells, B-cells, gamma delta T cells, cytotoxic cells, Th2 cells, CD8^+^ T cells and DC infiltrated less (Figure 2C).

### Identification of DE-mRNAs in cardiac tissue of patients with SCM

**Figure 3:**
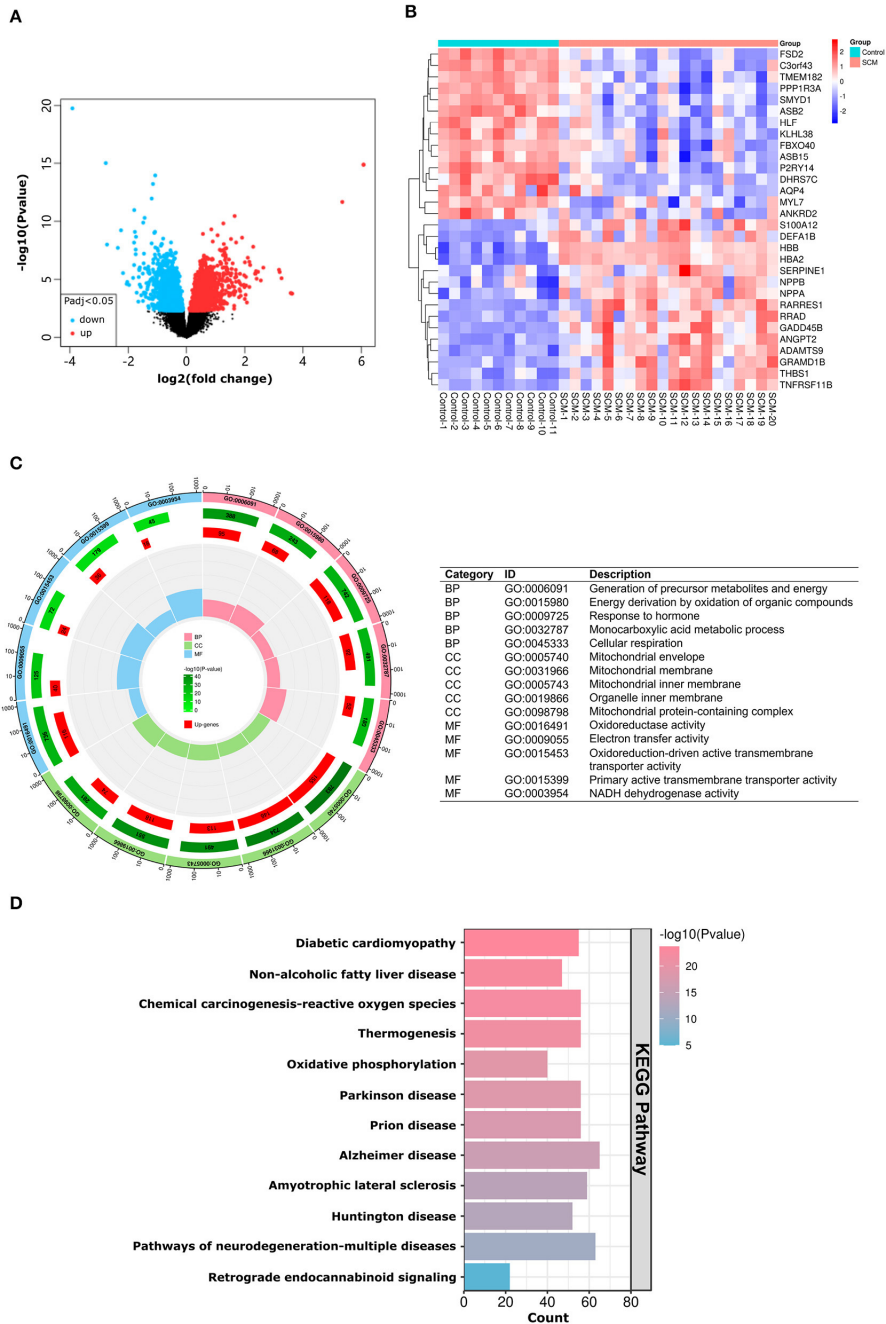
mRNA expression and DEGs enrichment analysis of dataset GSE79962. **(A)** Volcano plots corresponding to mRNA expression profiles in human hearts in the GSE79962 dataset. Red plots represent up-regulated mRNAs, black plots represent insignificant mRNAs, and blue plots represent down-regulated mRNAs. **(B)** Heatmap corresponding to the expression profile of the top 30 DE-mRNAs in human hearts in the GSE79962 dataset as determined by *p*-value. Red rectangles represent high expression and blue rectangles represent a low expression. **(C)** Circle plot showing the top 5 GO-enriched terms in BP, CC and MF. The most important BPs are involved in generation of precursor metabolites and energy, energy derivation by oxidation of organic compounds, and response to hormone; CC is involved in mitochondrial envelope, mitochondrial membrane and mitochondrial inner membrane; MF is involved in oxidoreductase activity, electron transfer activity, and oxidoreduction-driven active transmembrane transporter activity. **(D)** Histogram showing the most abundant KEGG pathway for DE-mRNA. The most important KEGG pathways are involved in diabetic cardiomyopathy, non-alcoholic fatty liver disease and chemical carcinogenesis-reactive oxygen species pathway. DE-mRNAs: Differentially expressed mRNAs; GO: Gene Ontology; BP: Biological Process; CC: Cellular Components; MF: Molecular Function; KEGG: Kyoto Encyclopedia of Genes and Genomes; The screening criteria for significantly enriched biological processes and pathways were *Q* < 0.05. *Q*-values are adjusted *p*-values.

A total of three datasets were used in this paper, and specific information about the datasets, including GEO accession number, experiment type, species, source tissue, number of samples from SCM and controls, and dataset functions are summarized in Table 1. A total of 34 SCM samples and 19 control samples from these three datasets were employed in this paper. There are 51 samples in the GSE79962 dataset, from which we extracted 20 cardiac mRNA array expression profiles of patients with septic cardiomyopathy and 11 cardiac mRNA array expression profiles of controls for analysis. The volcano plot depicts the expression distribution of all the genes in GSE79962 (Figure 3A). A total of 1592 DE-mRNAs (788 up-regulated and 804 down-regulated) were identified in GSE79962. The top 30 DE-mRNAs with the most significant differential changes (top 15 up-regulated and top 15 down-regulated) were then chosen for analysis and heatmap visualization of their inter-group expression differences (Figure 3B). GSE141864 and GSE72380 have 259 DE-mRNAs and 34 DE-miRNAs, respectively.

### Enrichment analysis of DE-mRNAs

According to the results of the GO analysis, these DE-mRNAs were linked to a variety of biological processes, molecular functions, and cellular components. The top three biological processes in which DE-mRNAs were involved were: generation of precursor metabolites and energy, energy derivation by oxidation of organic compounds, and response to hormone; the top three significant cellular components in which the DE-mRNAs participated were: mitochondrial envelope, mitochondrial membrane and mitochondrial inner membrane; the top three significant molecular functions related to those DE-mRNAs were: oxidoreductase activity, electron transfer activity, and oxidoreduction-driven active transmembrane transporter activity (Figure 3C). KEGG pathway analysis showed those DE-mRNAs to be significantly enriched in diabetic cardiomyopathy, non-alcoholic fatty liver disease and chemical carcinogenesis-reactive oxygen species pathway (Figure 3D). The results of GO/KEGG analysis of DE-mRNAs from Metascape online resources are shown in Supplementary Table S1.

### PPI network analysis and MCODE cluster modules

**Figure 4:**
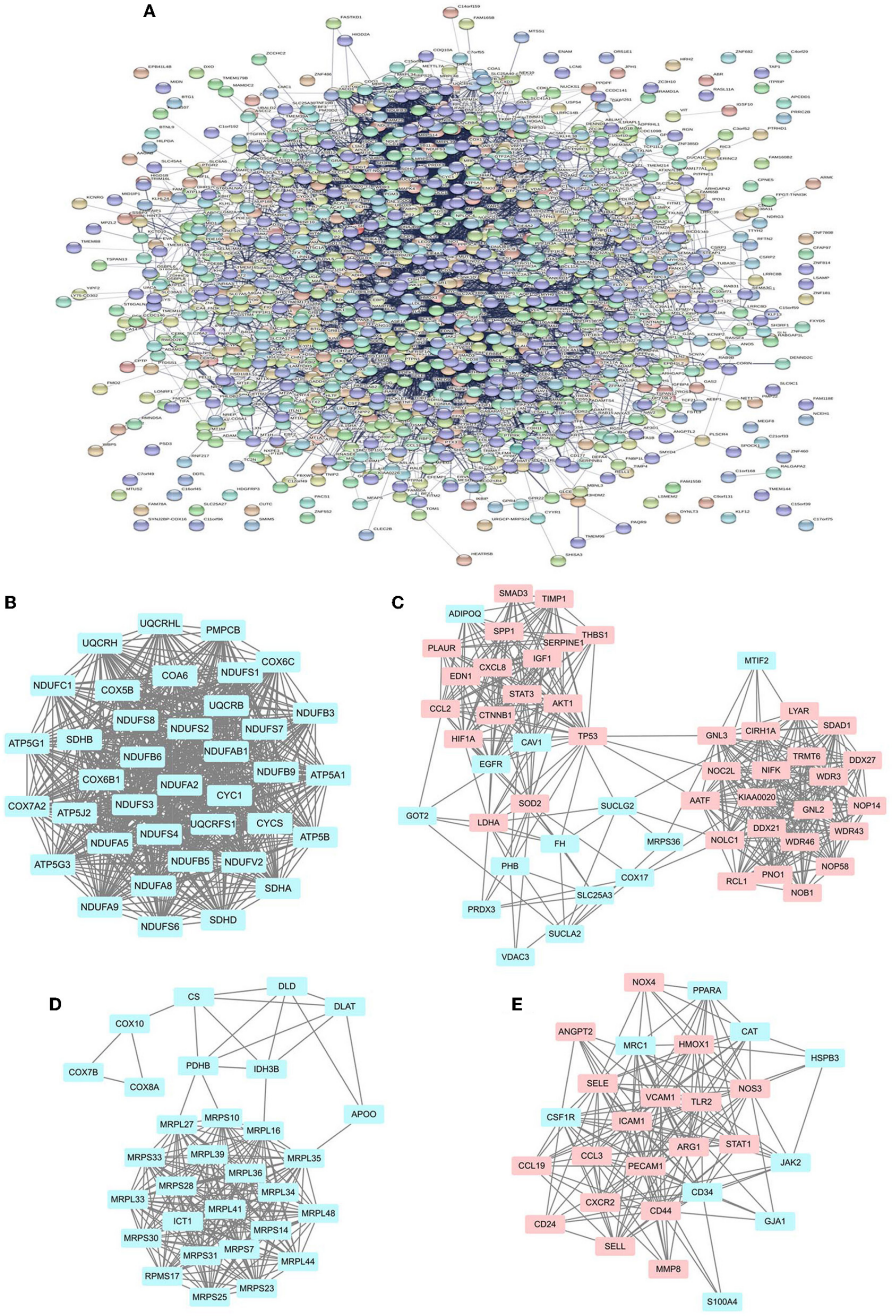
PPI network of DE-mRNAs and four cluster modules extracted by MCODE. **(A)** The interaction network between proteins encoded by DE-mRNA consists of 1,591 nodes and 12,507 edges. Each node represents a protein, and each edge represents a protein-protein association. The smaller the *Q-*value, the larger the shape size. **(B–E)** MCODE extracts four cluster modules. Cluster 1 had the highest cluster score (score: 36.811, 38 nodes and 681 edges), followed by cluster 2 (score: 16.235, 52 nodes, and 414 edges), cluster 3 (score: 15.862, 30 nodes, and 230 edges) and cluster 4 (score: 13.308, 27 nodes, and 173 edges). Blue indicates low expression of the gene, pink indicates high expression of the gene.

**Figure 5:**
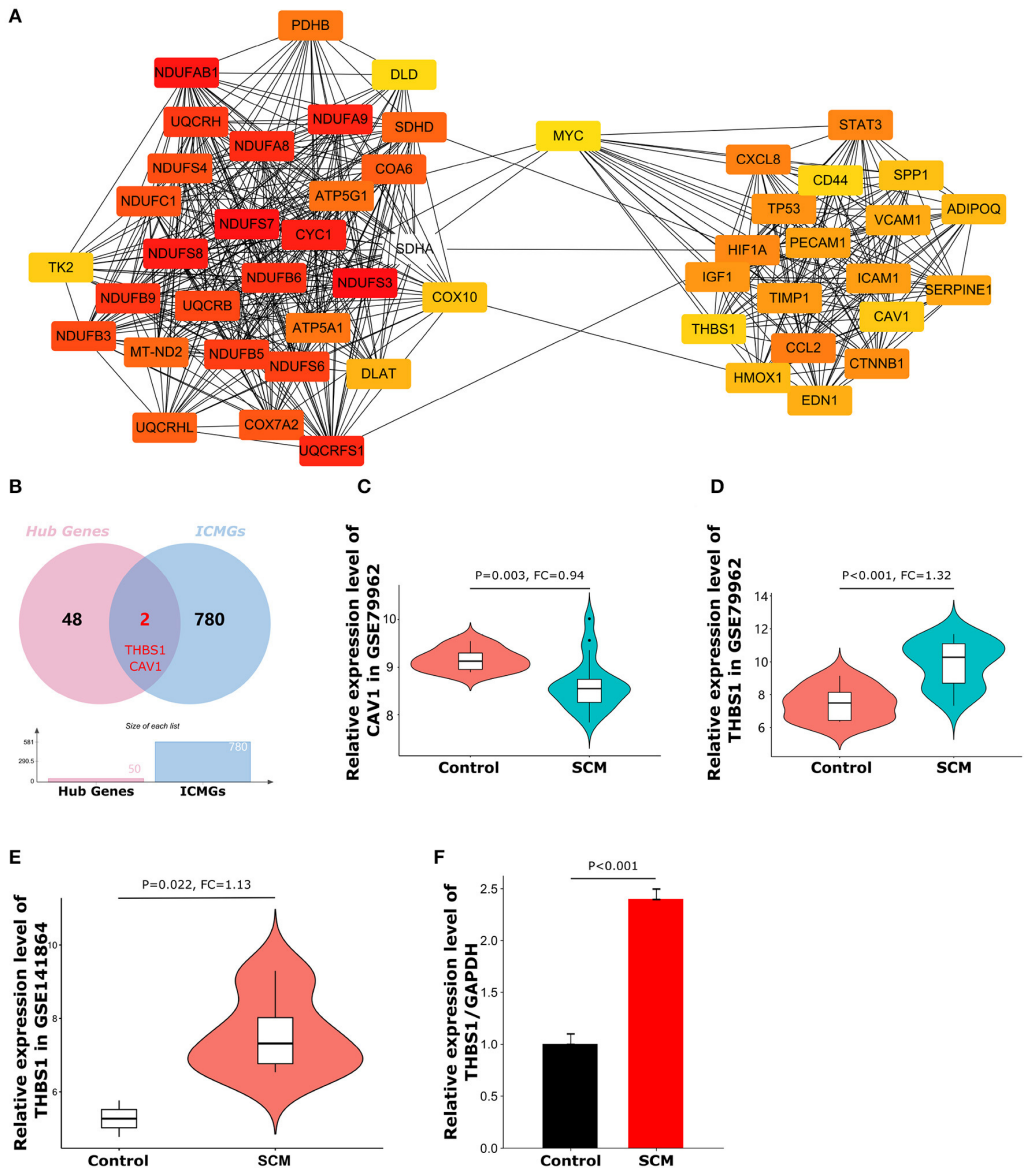
Top50 hub genes and expression of target genes. **(A)** Cluster plots represent the top 50 hub genes identified by CytoHubba. **(B)** Venn diagram of the intersection of hub genes and immune cell marker genes. **(C)** Expression levels of CAV1 in dataset GSE79962. **(D)** Expression levels of THBS1 in dataset GSE79962. **(E)** Expression levels of THBS1 in validation dataset GSE141864. **(F)** The expression level of THBS1 in peripheral blood mononuclear cells of SCM patients was detected by quantitative reverse transcription polymerase chain reaction.

The STRING online tool enables reciprocal analysis of differentially expressed genes and derives a PPI network containing network features and node information. The interaction network between proteins coded by DE-mRNAs, which was comprised of 1591 nodes and 12507 edges, was constructed by STRING and visualized (Figure 4A). The MCODE plugin was used to identify gene cluster modules. In this network, we identified four modules based on the filtering conditions, as shown in Figure 4. Cluster 1 had the highest cluster score (score: 36.811, 38 nodes and 681 edges) (Figure 4B), followed by cluster 2 (score: 16.235, 52 nodes, and 414 edges) (Figure 4C), cluster 3 (score: 15.862, 30 nodes, and 230 edges) (Figure 5D), and cluster 4 (score: 13.308, 27 nodes, and 173 edges) (Figure 4E). We then performed GO/KEGG analysis on cluster 1 and cluster 2 genes. The results showed that clusters 1 and 2 were mainly enriched in oxidative phosphorylation, ribosome biosynthesis and the HIF-1 signaling pathway, indicating that oxidation-related biological processes and pathways play an essential role in the pathogenesis of SCM. Meanwhile, the central position occupied by protein products in the PPI network suggests that genes in these clusters may be potential targets for SCM therapy. The results of GO/KEGG analysis of cluster 1 and cluster 2 are shown in Supplementary Table S2.

### Identification of CytoHubba hub genes and immune-related genes in SCM

CytoHubba is a plug-in that measures the importance of nodes in a network based on network features and provides 11 different topological analysis methods for biological network core element analysis. The Maximum Cluster Centrality (MCC) algorithm has better performance in predicting the accuracy of important proteins in the network. Therefore, the MCC algorithm was chosen in this study for the screening of focused key genes in the PPI network (22). To identify important genes in the PPI network, we used the CytoHubba plugin to identify hub genes. The top 50 hub genes (Figure 5A) were determined by the MCC algorithm on CytoHubba. Details of these hub genes are shown in Table 2. The hub gene refers to a gene that serves as a vital connection in the PPI network and may have a function in the regulation of SCM pathogenesis. Then, immune-related genes in SCM were also identified. We crossed 50 hub genes and 782 ICMGs and obtained 2 overlapping genes: CAV1 and THBS1 (Figure 5B). Figures 5C,D show the expression levels of CAV1 and THBS1 in dataset GSE79962. To obtain the target genes, we used the GSE141864 dataset as a validation dataset to verify whether 2 overlapping genes have a ubiquitous expression in SCM. Dataset GSE141864 validation results show that only THBS1 exists in both the dataset GSE141864 and the dataset GSE79962. And the expression of THBS1 in the GSE141864 dataset was upregulated (Figure 5E), which is consistent with the expression trend of THBS1 in the dataset GSE79962. Therefore, THBS1 was selected as the target gene for this study.

**Table 2:** 50 hub genes identified by MCC algorithms of CytoHubba.

**Gene symbol**	**Description**	**Log2FC**	***P*-value**	**Regulation**
NDUFS3	NADH: ubiquinone oxidoreductase core subunit S3	−1.737120445	0.0000114	Downregulated
NDUFS7	NADH: ubiquinone oxidoreductase core subunit S7	−1.574897275	0.00561	Downregulated
NDUFAB1	NADH: ubiquinone oxidoreductase subunit AB1	−2.542003558	0.00000009	Downregulated
NDUFS8	NADH: ubiquinone oxidoreductase core subunit S8	−1.579567009	0.0000371	Downregulated
CYC1	Cytochrome c1	−2.076895721	0.00000486	Downregulated
NDUFA9	NADH: ubiquinone oxidoreductase subunit A9	−2.266993522	0.00000073	Downregulated
UQCRFS1	Ubiquinol-cytochrome c reductase, Rieske iron-sulfur polypeptide 1	−1.705705436	0.000108	Downregulated
NDUFA8	NADH: ubiquinone oxidoreductase subunit A8	−2.482463744	0.00000684	Downregulated
NDUFB6	NADH: ubiquinone oxidoreductase subunit B6	−2.039390389	0.0000192	Downregulated
NDUFB9	NADH: ubiquinone oxidoreductase subunit B9	−2.014906417	0.00000284	Downregulated
UQCRH	Ubiquinol-cytochrome c reductase hinge protein	−3.111336722	0.0000538	Downregulated
NDUFB5	NADH: ubiquinone oxidoreductase subunit B5	−2.941401697	0.00000012	Downregulated
NDUFS6	NADH: ubiquinone oxidoreductase subunit S6	−1.564226777	0.000194	Downregulated
UQCRB	Ubiquinol-cytochrome c reductase binding protein	−2.265991794	0.0000501	Downregulated
NDUFB3	NADH: ubiquinone oxidoreductase subunit B3	−2.914753787	0.00000001	Downregulated
NDUFS4	NADH: ubiquinone oxidoreductase subunit S4	−1.666024407	0.000942	Downregulated
NDUFC1	NADH: ubiquinone oxidoreductase subunit C1	−1.694958833	0.0000612	Downregulated
COX7A2	Cytochrome c oxidase subunit 7A2	−1.691922457	0.0000896	Downregulated
COA6	Cytochrome c oxidase assembly factor 6	−1.844743374	0.000682	Downregulated
UQCRHL	Ubiquinol-cytochrome c reductase hinge protein like	−2.243260438	0.00000231	Downregulated
SDHD	Succinate dehydrogenase complex subunit D	−1.899544757	0.0000576	Downregulated
SDHA	Succinate dehydrogenase complex flavoprotein subunit A	−1.848489446	0.000278	Downregulated
PDHB	Pyruvate dehydrogenase E1 subunit beta	−2.406816797	0.00000013	Downregulated
CCL2	C-C motif chemokine ligand 2	5.98480273	0.000357	Upregulated
CXCL8	C-X-C motif chemokine ligand 8	2.48555523	0.0337	Upregulated
STAT3	Signal transducer and activator of transcription 3	3.798381512	0.00000003	Upregulated
HIF1A	Hypoxia-inducible factor 1 subunit alpha	2.044141975	0.0046	Upregulated
CTNNB1	Catenin beta 1	1.617683709	0.000253	Upregulated
TP53	Tumor protein p53	2.528141584	0.00000383	Upregulated
IGF1	Insulin-like growth factor 1	3.239931582	0.00158	Upregulated
TIMP1	TIMP metallopeptidase inhibitor 1	4.349271796	0.000184	Upregulated
ICAM1	Intercellular adhesion molecule 1	2.807022297	0.00599	Upregulated
PECAM1	Platelet and endothelial cell adhesion molecule 1	2.089522968	0.00000645	Upregulated
SERPINE1	Serpin family E member 1	6.890206457	0.000999	Upregulated
VCAM1	Vascular cell adhesion molecule 1	1.83948928	0.0182	Upregulated
EDN1	Endothelin 1	1.946877881	0.0126	Upregulated
DLAT	Dihydrolipoamide S-acetyltransferase	−2.103045773	0.00000176	Downregulated
ADIPOQ	Adiponectin, C1Q and collagen domain containing	−3.441462229	0.000137	Downregulated
HMOX1	Heme oxygenase 1	3.518726822	0.00898	Upregulated
SPP1	Secreted phosphoprotein 1	5.222682665	0.0105	Upregulated

### Validation of target genes

To further verify the reliability of the expression level of the target gene THBS1, we collected PBMCs from three clinical SCM patients and three normal individuals for qRT-PCR experiments. The primer sequences and other characteristics used for qRT-PCR analysis are shown in Table 3. As shown in Figure 5F, the expression level of THBS1 in PBMCs of SCM patients was significantly higher than that of normal controls (*P* < 0.01).

**Table 3:** Primer pairs for qRT-PCR used in this study.

**Target**	**Primer sequences, 5^′^-3^′^**	**Product size (bp)**
THBS1	Forward: AAGGACTGCGTTGGTGATGT Reverse: AGCTAGTACACTTCACGCCG	109
miR-222-3p	Forward: GAGCTACATCTGGCTACTGGGTAA Reverse: GCGAGCACAGAATTAATACGAC	24

### IRHG-THBS1 has excellent diagnostic efficacy for SCM

**Figure 6:**
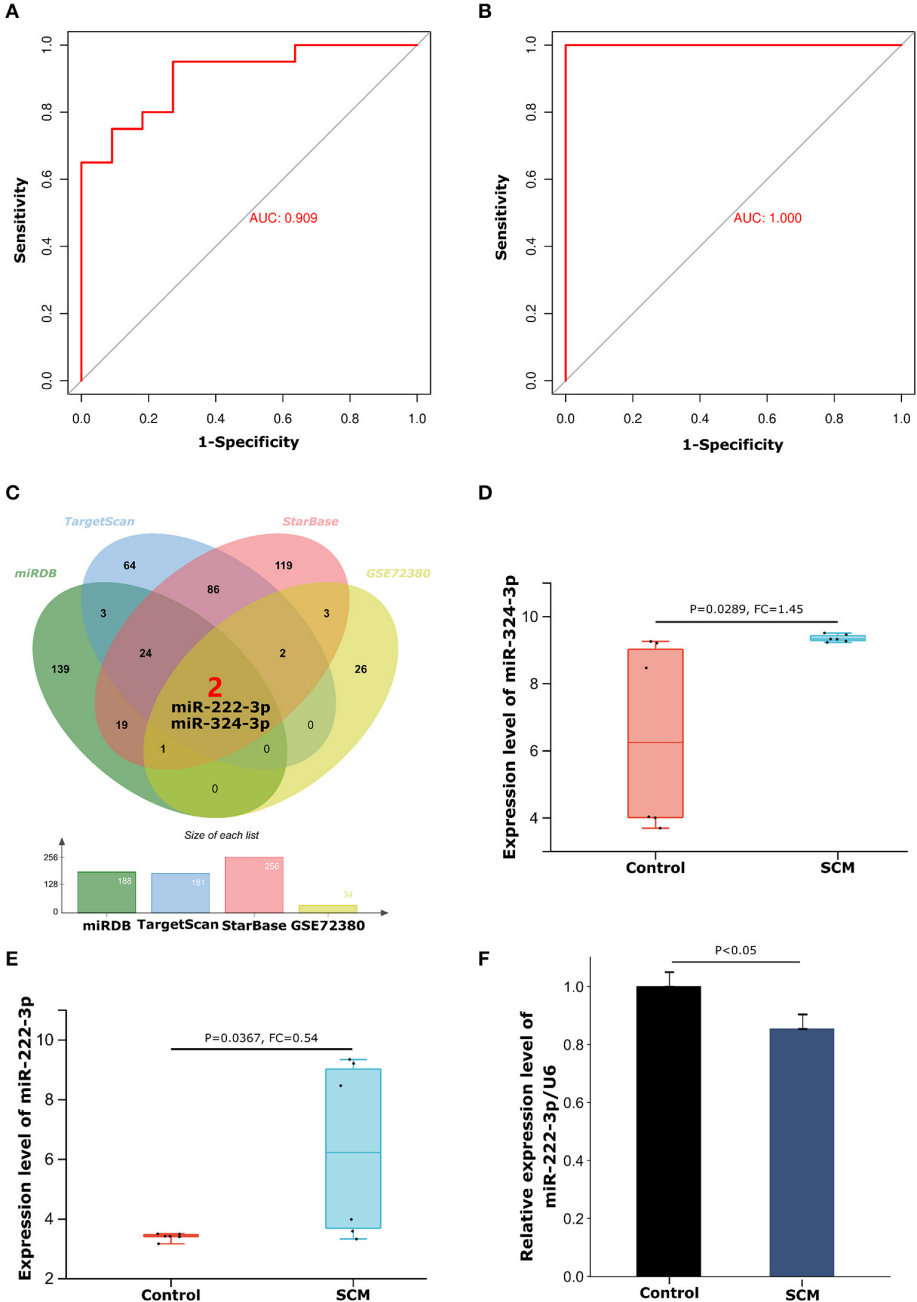
ROC curve diagnostic results of THBS1 and construction of the miRNA-mRNA regulatory network. **(A)** ROC curve of THBS1 in test dataset GSE79962. **(B)** ROC curve of THBS1 in validation dataset GSE14186. **(C)** Venn diagram of the intersection of THBS1's miRDB, TargetScan and StarBase database prediction results and GSE72380 dataset. **(D)** Expression levels of miR-324-3p in the GSE72380 dataset. **(E)** Expression levels of miR-222-3p in the GSE72380 dataset. **(F)** The expression level of miR-222-3p in peripheral blood mononuclear cells of SCM patients was detected by quantitative reverse transcription polymerase chain reaction.

ROC curve analysis further demonstrated that THBS1 has a good role in discriminating SCM patients from healthy controls in the experimental dataset GSE79962 and the validation dataset GSE141864. The results showed that the area under the ROC curve (AUC) for the experimental dataset GSE79962 was 0.909 (Figure 6A), and the AUC for the validation dataset GSE141864 was 1.000 (Figure 6B). This indicates that THBS1 has a good ability to distinguish SCM patients from healthy controls, suggesting that THBS1 has potential diagnostic value for septic cardiomyopathy.

### Immune-related miR-222-3p/THBS1 network in SCM

From the miRDB, TargetScan and StarBase databases, we predicted 188, 181, and 256 miRNAs, respectively Details of the prediction results are shown in the Supplementary Table S3. We then took the intersecting miRNAs from the prediction results of the three databases and validated them using the GSE72380 dataset, resulting in 2 shared miRNAs: miR-324-3p and miR-222-3p (Figure 6C). The expression levels of miR-324-3p (*P* = 0.0289, FC = 1.45) and miR-222-3p (*P* = 0.0367, FC = 0.54) in the GSE72380 dataset are shown in Figures 6D,E. Functional studies of miRNAs show that miRNAs often act as mRNA sponges in the process of transcript regulation, inhibiting mRNA expression or promoting mRNA degradation, that is, miRNAs are negatively correlated with mRNA levels (30). Therefore, based on this mechanism of action of miRNAs, we selected the down-regulated miR-222-3p as the regulatory miRNAs of THBS1. Concomitantly, THBS1 was similarly predicted in the results of the RNA22 database for reverse prediction of target genes of miR-222-3p, which further enhanced the reliability of the results. The target gene prediction results of miR-222-3p are shown in Supplementary Table S4. Next, to verify the expression of miR-222-3p in SCM patients, PBMCs from SCM patients and healthy controls were extracted for qRT-PCR. The results showed that miR-222-3p was lowly expressed in PBMCs of SCM patients, which was consistent with the miRNA-mRNA regulatory hypothesis (Figure 6F). Thus, we finally obtained the miR-222-3p/THBS1 regulatory network with a regulatory role in SCM.

## Discussion

The occurrence of septic cardiomyopathy is very detrimental to the survival and prognosis of septic patients. At the same time, the multiple organ chain reaction and circulatory system failure of SCM have also attracted scholars to study SCM. Currently, systolic or diastolic ventricular dysfunction and primary cardiomyocyte injury are commonly used as clinical criteria for the evaluation of SCM. However, the commonly used SCM diagnosis based on echocardiography (31), B-type natriuretic peptide (BNP) (32) and cardiac troponin I (cTnI) and other markers of myocardial injury have poor sensitivity and specificity, hindering early intervention in SCM. Therefore, finding more sensitive and specific biomarkers in SCM is crucial for early intervention in SCM.

With the development of genomics and the continuous maturation of gene sequencing technology, target gene technology is also increasingly used for disease localization, early diagnosis and treatment (33, 34). The construction of gene co-expression networks is an important direction of current disease research. By analyzing the changes of gene expression under pathological conditions, we can explore the key genes and mechanisms of disease development, reveal the changes of disease gene level, and provide direction for early diagnosis of disease (35).

The balance of the immune system plays an important role in maintaining the body's homeostasis, and an excessive or unregulated immune response will cause severe organ/tissue damage in the body. Existing studies suggest that the immune microenvironment plays an important role in the occurrence, progression, and susceptibility of sepsis (36, 37). For example, in the pathogenesis of SCM, it has been found that the activation of the complement system (38) and the increase of extracellular histones (39, 40) can further deteriorate the function of cardiomyocytes. Therefore, immune response may play an important regulatory role in SCM. However, the specific mechanism of action of IRGs in SCM remains unclear. Therefore, we aimed to analyze the SCM dataset by bioinformatics methods to explore the regulatory role of IRGs and their co-expression networks in SCM.

Microarray dataset analysis based on public databases is currently an effective method in the screening of disease target genes (41). The GSE79962 and GSE141864 datasets collected myocardial tissue samples from patients who died of sepsis and controls who died from sepsis and performed an array of data. Both datasets are well-organized and include abundant sample sizes, producing them good resources for bioinformatics analysis. In this study, we integrated the septic cardiomyopathy dataset with 782 immune cell marker genes (ICMGs), applied bioinformatics approaches to identify IRHGs with immunomodulatory roles in human SCM, and constructed immune-related miRNA-IRHGs regulatory axis in SCM, with the intention of improving our understanding of SCM pathogenesis and exploring its potential biomarkers. The dataset GSE79962 is used to investigate critical genes and mechanisms of SCM in this paper. To clarify the differences in immune cell composition between SCM myocardial tissue and normal myocardial tissue, we performed an immune cell infiltration analysis of the dataset GSE79962. The results of the analysis showed that there were 11 types of immune cells with significant differences in the degree of infiltration between SCM myocardial tissue and normal tissue. Th17 cells play an important role in the regulation of immune-related diseases (42), and previous findings suggest that an increase in neutrophils and Th17 cells may lead to a decrease in immune system defenses in patients with sepsis (43, 44); Studies on Tfh cells have shown that Tfh cells are associated with mortality warnings in patients with sepsis, and low Tfh cell levels may imply a poor prognosis for patients (45); Danahy et al. 's research shows that in sepsis, not only the total number of CD8^+^ T cells decreases, but also the antigen-driven proliferation capacity and effector function of CD8^+^ T cells are also impaired (46); These are consistent with the results of our immune infiltration analysis, which further confirmed the important role of abnormal immune response in the pathogenesis and progression of SCM.

After analyzing the immune infiltration of dataset GSE79962, DE-mRNAs of dataset GSE79962 were further screened and subjected to GO/KEGG analysis. A total of 788 up-regulated and 804 down-regulated SCM-related DE-mRNAs were identified in the GSE79962 dataset. And these DE-mRNAs are related to biological processes such as the generation of precursor metabolites and energy, energy derivation by oxidation of organic compounds, and response to hormone. To further screen genes with increasing regulatory roles in SCM, a PPI network based on DE-mRNAs in the STRING public database was constructed. Through the PPI network, we obtained 4 central clusters and the top 50 hub genes in DE-mRNAs. After crossing the Top 50 hub genes with ICMGs and validated by the GSE141864 dataset and qRT-PCR, the central target gene THBS1 with immune function in SCM was finally obtained. The ROC curve is an important step in the development of a test with the desired level of sensitivity and specificity (47) and is important in assessing whether a given test marker has a valid diagnostic ability in the early stages of the disease (26). The AUC values under the ROC curve of THBS1 were 0.909 and 1.000, indicating that THBS1 has a good ability to distinguish SCM patients from healthy people.

THBS1 (thrombospondin 1), a member of the thrombospondin family, is expressed in many tissues during embryonic development, but in healthy adults, it is associated with ischemia-reperfusion, tissue remodeling, immune system damage (rheumatoid synovium, glomerulonephritis), atherosclerotic lesions, and high glucose and high-fat induction. Xie et al.'s model of LPS-induced sepsis showed that THBS1 was also high in injured cardiomyocytes, accompanied by increased rates of inflammatory cytokines, ROS and apoptosis, indicating that THBS1 has a regulatory role in the inflammatory response of SCM, which coincides with our result that THBS1 is an immune gene (48); McMaken et al. showed that THBS1–/– mouse macrophages had stronger phagocytosis, increased bacterial clearance, indicating that THBS1 can be activated by innate Sexual immunity affects mortality in SCM mice (49); in addition, thrombocytopenia during sepsis is also associated with THBS1 (50); Sun et al. showed that knockdown of THBS1 level can inhibit the TGF-β/Smad3 pathway and alleviate sepsis-induced pyroptosis (51). These suggest that IRCGs-THBS1 is likely to modulate the development of SCM through ROS oxidative stress, enhanced macrophage clearance, or TGF-β-related pathways, providing research directions for the treatment of SCM and suggesting that THBS1 levels have high diagnostic value for SCM detection.

In addition, although the immune response is an important pathological process in SCM, immune-related regulatory networks in SCM are uncommon. Here, we construct a miRNA-IRHGs regulatory network for THBS1 to explore the potential mechanism of action of THBS1 in SCM immunity. In this study, the immune-related miR-222-3p/THBS1 signal axis in SCM was identified by bioinformatics analysis.

### miR-222-3p/THBS1 regulatory axis

MicroRNAs have regulatory roles at both the transcriptional and post-transcriptional levels of gene information, and according to the mRNA repression effect of miRNAs (52), the level of target miRNAs should be negatively correlated with THBS1. Our results show that IRHGs-THBS1 is upregulated in SCM. This means that miRNAs upstream of IRHG-THBS1 should be at low levels in the SCM state. The LPS-induced SCM model mouse heart dataset GSE72380 was used as a validation dataset in this paper. Data from mouse myocardial tissue and *in vivo* expression data will demonstrate the stability and reliability of the experimental results. Finally, through database prediction, dataset GSE72380 and qRT-PCR validation, the down-regulated miR-222-3p in SCM was selected as the target miRNA.

Xu et al. performed miRNA array analysis on the plasma of septic mice and showed that the level of miR-222-3p in extracellular vesicles (EVs) was more than 1.5-fold higher than that in control mice, and miR-222-3p regulates cytokine production through downstream TLR7-MyD88 signaling, affecting SCM myocardial inflammation (53); However, the current research on miR-222-3p in sepsis and septic cardiomyopathy is scarce, but other scholars have studied the research of miR-222-3p in rheumatoid arthritis (54) and hepatitis (55), which indirectly reflects the inflammatory regulation of miR-222-3p and provides a reference for the follow-up research of miR-222-3p in SCM. Sun et al.'s (56) study on liver cancer showed that miR-222-3p could regulate the expression of THBS1 and affect the proliferation and apoptosis of hepatocellular carcinoma. However, there is a lack of research on the miR-222-3p expression levels in SCM cardiomyocytes and the mutual regulation of miR-222-3p with THBS1, which means that we still need to conduct further cellular or in vivo SCM experiments to verify them.

However, SCM is a complex immune system disease caused by pathogen invasion and immune system activation. Furthermore, multi-organ damage is involved in the development of SCM, which means that diagnostic markers for SCM are abundant and variable. Our findings demonstrate that THBS1, which is highly expressed in SCM cardiomyocytes, may be an important biomarker for SCM diagnosis. Compared with myocardial injury markers and inflammation-associated proteins with diagnostic support for SCM, such as troponin (cTnI, cTnT), B-type NP (BNP) (57) and inflammation-associated monocyte chemotactic protein 1 (MCP-1) (58), THBS1 identified in this study is not only elevated in human SCM cardiac tissue but also has miRNA regulatory mechanisms upstream of it. This further illustrates the sensitivity and diversity of THBS1 regulation. However, some scholars' studies have shown that THBS1 is involved not only in SCM, but also in atrial fibrillation (59) and tumor invasion (60), therefore, an in-depth exploration of the diagnostic and regulatory ability of THBS1 and its miR-222-3p/THBS1 signaling pathway in SCM, and testing whether THBS1 can be used as a stable biomarker for SCM diagnosis independent of other diseases, will be more home clarify the perspective of this study and provide the best possible de support for early clinical intervention and treatment of SCM.

From the published articles, we also found that Chen et al. (61), Kang et al. (62), Gong et al. (63), Li et al. (64), and Wang et al. (65). similarly applied bioinformatics approaches to study the hub genes in SCM. First and foremost, our studies with Chen et al. (61), Kang et al. (62), Li et al. (64), and Wang et al. (65) used the human SCM dataset GSE79962 as an experimental dataset, but we innovatively combined immune cell marker genes with SCM differentially expressed genes to identify the biomarker role of IRHG in SCM; Secondly, we also used MCODE to identify the central clusters of SCM differentially expressed genes to explore modules that play an important interaction in SCM and their involved pathways; Thirdly, different from the studies of Kang et al. (62) and Gong et al. (63), we also extracted human samples to verify the expression of target genes, and used ROC curves to analyze the diagnosticity of target genes for SCM. Finally, a miRNA-IRHG pair that is different from previous studies and has an immunomodulatory role in SCM was obtained: miR-222-3p/THBS1. Details of the comparison between this study and previous studies are presented in Table 4.

**Table 4:** The innovations in this study compared to what has been published.

**Items**		**Identification of hub genes in SCM based on bioinformatics analyses**				
	**Our findings**	**Cell paper** **(PMID: 31424269)**	**Cell paper** **(PMID: 31794266)**	**Cell paper** **(PMID: 31794266)**	**Cell paper** **(PMID: 35003233)**	**Cell paper** **(PMID: 32147601)**
Publication date	–	2019	2020	2022	2021	2020
Test datasets	GSE79962	GSE79962	GSE79962	GSE63920 and GSE44363	GSE79962	GSE79962 and GSE53007
Tissue/species	Heart/human	Heart/human	Heart/human	Heart/mice	Heart/human	Heart/human/mouse
Immune infiltration analysis	Applied	–	–	–	Applied	–
Gene clusters	Applied	–	–	–		
Hub genes	Immune-related hub gene**:** THBS1	MYC, SERPINE1, CCL2, STAT3	NDUFB5, TIMMDC1, VDAC3	FRGs: Cdkn1a, Ptgs2, Nfe2l2, Rela and Vim	LRRC39,COQ10A, FSD2, PPP1R3A, TNFRSF11B, IL1RAP, DGKD, POR and THBS1	CCL2,TIMP-1,SOCS3 and IL1R2
Dry validation	GSE141864 and GSE72380	–	–	GSE72380 and GSE29914	—	—
Wet verification	Human PBMCs: qRT-PCR	–	Mice heart: WB	Mice: RT-PCR	–	Human blood: ELISA
ROC curves	Applied	–	–	–	Applied	Applied
Mechanism	miRNA-IRHG pair: miR-222-3p/THBS1	TF-miRNA-mRNA network	–	miR-1892/Cdkn1a pair	–	–

IRHG, Immune-related hub gene; *FRGs*, Ferroptosis-related genes; *PBMCs*, Peripheral blood mononuclear cells; *WB*, Western Blot; *TF*, Transcription factor; ELISA, enzyme-linked immunosorbent assay.

**Figure 7:**
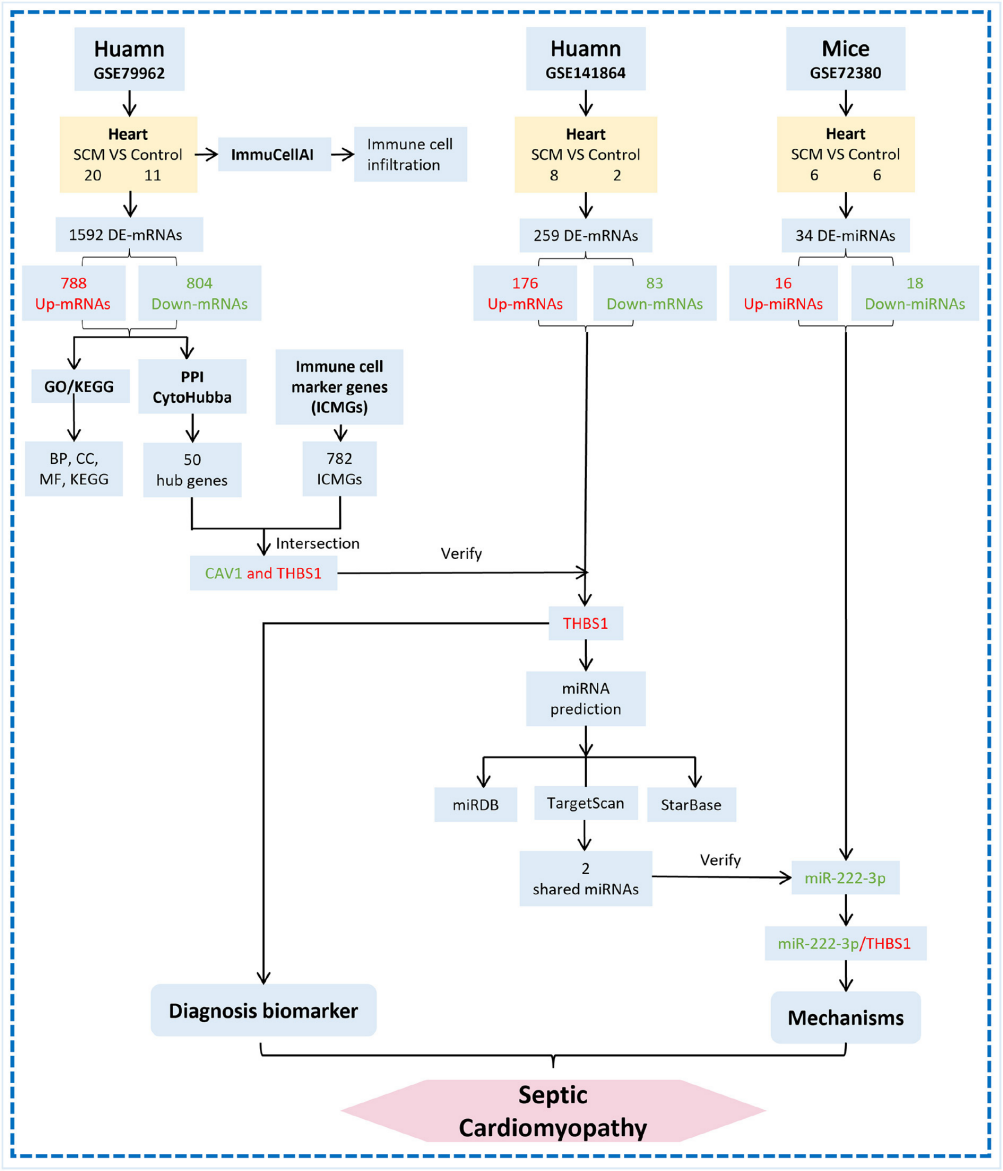
Flow chart of miRNA-mRNA regulatory network construction in cardiac tissue of SCM patients. The red word means upregulation and the green word means downregulation.

In summary, this study used bioinformatics analysis to innovatively combine the GEO database with immune cell marker genes to investigate the presence of immune-related diagnostic biomarkers THBS1 in infectious cardiomyopathy and to establish miR-222-3p/THBS1 regulatory pairs with immunomodulatory effects. The findings of this work demonstrate the biological regulatory role of epigenetic mechanisms in the progression of SCM and analyze important biomarkers and prospective immunotherapeutic targets for SCM, providing new therapeutic targets and directions for early intervention in SCM. However, studies on the epigenetic mechanisms of SCM are still lacking. The acquisition of myocardial tissues from SCM patients and the completion of the identification of SCM differentially expressed genes and their epigenetic mechanisms by gene sequencing technology will advance the realization of early clinical intervention in SCM and deepen our understanding of the pathogenesis of septic cardiomyopathy. The new working model used in this study on human SCM research is shown in Figure 7.

However, it is worth noting that there are still some restrictions on our study. First, bioinformatics analysis was the main method of this study, and we next need to construct an experimental animal model of SCM to study the expression level of miR-222-3p/THBS1 and its regulatory mechanism *in vivo*; second, although 19 control samples and 34 SCM samples have been used for the evaluation and experimental validation of this study, further expansion of the study sample size will enhance the credibility of our results. Additionally, only PBMCs from SCM patients were collected for the outcome validation of this study, and the sample size of PBMCs was not large enough, which may cause information bias to some extent. The further acquisition of more peripheral blood and even myocardial tissues from SCM patients for gene expression analysis would improve the credibility and authenticity of the results of this study. Last but not least, the dearth of intrinsic information regarding the level of SCM activity necessitates further analysis of the relationship between immune genes and their regulatory networks and disease and disease severity.

## Data availability statement

Publicly available datasets were analyzed in this study. This data can be found at: https://www.ncbi.nlm.nih.gov/gds/?term$=$GSE79962; https://www.ncbi.nlm.nih.gov/gds/?term=GSE141864; https://www.ncbi.nlm.nih.gov/gds/?term=GSE72380.

## Ethics statement

The studies involving human participants were reviewed and approved by Ethics Committee of the First Affiliated Hospital of Kunming Medical University, Yunnan Province [(2022), ethical review L, No. 23]. The patients/participants provided their written informed consent to participate in this study.

## Author contributions

All authors listed have made a substantial, direct, and intellectual contribution to the work and approved it for publication.

## Funding

This study was supported by the National Natural Science Foundation of China (Nos. 81860073 and 81760074), Special Foundation Projects of Joint Applied Basic Research of Yunnan Provincial Department of Science and Technology with Kunming Medical University [No. 2019FE001(-138)], Yunnan Provincial Department of Science and Technology (No. 202001AT070039), Yunnan Health Training Project of High Level Talents (No. H-2018032), 100 Young and Middle-aged Academic and Technical Backbones of Kunming Medical University (No. 60118260106), Young Talents of Yunnan Thousand Talents Plan (RLQN20200002), and Clinical Medcial Center for Cardiovascular and Cerebrovascular Disease of Yunnan Province (No. ZX2019-03-01).

## Conflict of interest

The authors declare that the research was conducted in the absence of any commercial or financial relationships that could be construed as a potential conflict of interest.

## Publisher's note

All claims expressed in this article are solely those of the authors and do not necessarily represent those of their affiliated organizations, or those of the publisher, the editors and the reviewers. Any product that may be evaluated in this article, or claim that may be made by its manufacturer, is not guaranteed or endorsed by the publisher.
